# The Italian arm of the PREPARE study: an international project to evaluate and license a maternal vaccine against group B streptococcus

**DOI:** 10.1186/s13052-020-00923-3

**Published:** 2020-10-28

**Authors:** Alberto Berardi, Tiziana Cassetti, Roberta Creti, Caterina Vocale, Simone Ambretti, Mario Sarti, Fabio Facchinetti, Stephen Cose, the Prepare Network, Merijn van Bijlsma, Diederik van De Beek, Claire Poyart, Neil French, Maryke Nielsen, Philippa Musoke, Kirsty Le Doare, Paul Heath, Hannah Davies, Sara Ovale, Licia Lugli, Maria Grazia Capretti, Marcello Lanari, Arianna Dondi, Matilde Ciccia, Rosa Francavilla, Angela Lanzoni, Lorenza Baroni, Sara Fornaciari, Edoardo Carretto, Cristina Alessandrini, Gambini Lucia, Serafina Perrone, Adriana Calderaro, Pierluigi Bacchini, Chiara Giugno, Claudio Rota, Rossella Pagano, Battista Guidi, Giacomo Biasucci, Belinda Benenati, Roberta Schiavo, Giancarlo Piccinini, Rita Pulvirenti, Vittoria Rizzo, Gina Ancora, Chiara China, Irene Papa, Laura Viola, Maria Federica Pedna, Jenny Bua, Laura Travan, Marina Busetti, Daniele Santori, Daniele Merazzi, Angela Papa, Ligi Laura, Cinzia Auriti, Paola Bernaschi, Giovanni Vento, Lucia Giordano, Teresa Spanu, Cristina Haass, Maria Carmela Margiotta, Giovanna Nardella, Rosella De Nittis, Nicola Laforgia, Sabrina Loprieno, Latorre Giuseppe, Angela Maria Moramarco, Chryssoula Tzialla, Valeria Fasolato, Silvia Orlandini, Lidia Decembrino, Giulia Del Campo, Angela Maiocchi, Armando Cuttano, Cristina Tuoni, Simona Barnini, Virgilio Carnielli, Barbara Perrone, Francesca Orecchioni, Federica Visintini, Alessandra Arzese, Paul Heath, Kirsty Le Doare

**Affiliations:** 1 Unità Operativa di Terapia Intensiva Neonatale, Dipartimento Integrato Materno-Infantile, Azienda Ospedaliero-Universitaria Policlinico, Via del Pozzo, 71, 41124 Modena, Italy; 2 Unità Operativa di Microbiologia Clinica, Azienda Ospedaliero- Universitaria Policlinico, Modena, Italy; 3 Reparto di Antibiotico Resistenza e Patogeni Speciali (AR-PS), Dipartimento di Malattie Infettive, Istituto Superiore di Sanità, Rome, Italy; 4 Unità Operativa di Microbiologia Clinica, Centro di Riferimento Regionale per le Emergenze Microbiologiche, (CRREM), Policlinico S. Orsola-Malpighi, Università di Bologna, Bologna, Italy; 5 Unità Operativa di Microbiologia, Azienda Ospedaliero-Universitaria S. Orsola-Malpighi, Bologna, Italy; 6 Department of Medical and Surgical Sciences for Mother, Child and Adult, University of Modena and Reggio Emilia, Azienda Ospedaliero Universitaria Policlinico, Modena, Italy; 7 MRC/UVRI and LSHTM Uganda Research Unit, Entebbe, Uganda; 8 Department of Clinical Research, LSHTM, London, UK; 9 St George’s Vaccine Institute, Institute of Infection and Immunity, St George’s, University of London, London, UK; 10 Paediatric Infectious Diseases Research Group, St George’s University of London, London, UK

**Keywords:** Group B *streptococcus*, Vaccine, Newborn, Sepsis, Meningitis, Prevention

## Abstract

**Background:**

Group B streptococcus (GBS) is a leading cause of sepsis, pneumonia and meningitis in infants, with long term neurodevelopmental sequelae. GBS may be associated with poor pregnancy outcomes, including spontaneous abortion, stillbirth and preterm birth. Intrapartum antibiotic prophylaxis (IAP) is currently the only way to prevent early-onset disease (presenting at 0 to 6 days of life), although it has no impact on the disease presenting over 6 days of life and its implementation is challenging in resource poor countries. A maternal vaccine against GBS could reduce all GBS manifestations as well as improve pregnancy outcomes, even in low-income countries.

**Main body:**

The term “PREPARE” designates an international project aimed at developing a maternal vaccination platform to test vaccines against neonatal GBS infections by maternal immunization. It is a non-profit, multi-center, interventional and experimental study (promoted by the St George University of London. [UK]) with the aim of developing a maternal vaccination platform, determining pregnancy outcomes, and defining the extent of GBS infections in children and mothers in Africa. PREPARE also aims to estimate the protective serocorrelates against the main GBS serotypes that cause diseases in Europe and Africa and to conduct two trials on candidate GBS vaccines.

PREPARE consists of 6 work packages. In four European countries (Italy, UK, Netherlands, France) the recruitment of cases and controls will start in 2020 and will end in 2022. The Italian PREPARE network includes 41 centers. The Italian network aims to collect: GBS isolates from infants with invasive disease, maternal and neonatal sera (cases); cord sera and GBS strains from colonized mothers whose infants do not develop GBS infection (controls).

**Short conclusion:**

PREPARE will contribute information on protective serocorrelates against the main GBS serotypes that cause diseases in Europe and Africa. The vaccine that will be tested by the PREPARE study could be an effective strategy to prevent GBS disease.

## Background

Group B streptococcus (GBS or *Streptococcus agalactiae*) is a Gram-positive pathogen belonging to Lancefield group B. It is a common commensal of the gastrointestinal tract and colonizes 10–30% of pregnant women at vaginal or vaginal/rectal sites [1]. In pregnant women, GBS is a frequent causative agent in urinary tract infections, chorioamnionitis and postpartum endometritis, and it is also associated with poor pregnancy outcomes, including spontaneous abortion, stillbirth and preterm birth [2].

GBS is a leading cause of sepsis, pneumonia and meningitis in infants, with long-term neurodevelopmental sequelae. Neonatal GBS infections are usually divided into Early-Onset Disease (EOD, presenting at 0 to 6 days of life) and Late-Onset Disease (LOD, presenting at 7 to 90 days of life) [3]. EOD is prevented through intrapartum antibiotic prophylaxis (IAP) in women with GBS colonization or obstetrical risk factors for GBS vertical transmission.

## Main text

The term “PREPARE” designates an international project entitled “Prevention of invasive Group B Streptococcus disease in young infants: a pathway for the evaluation & licensure of an investigational maternal GBS vaccine”. PREPARE is aimed at developing a vaccine against neonatal GBS infections and is promoted by the St George University of London (UK) (see https://gbsprepare.org).

This project is part of the EDCTP2 program (European & Developing Countries Clinical Trials Partnership) that funds research for prevention and treatment of poverty-related infectious diseases in sub-Saharan Africa and it is aligned with the WHO roadmap [1]. Moreover, PREPARE is supported by Horizon 2020 (European Union’s Framework Program for Research and Innovation).

Strategies implementing IAP, especially those that screen women in late pregnancy for vagino-rectal GBS colonization (regardless of presenting risk factors) has led to a dramatic decline in the incidence rates of EOD (i.e., from 0.37 to 0.22 per 1000 live births from 2006 to 2017 in USA) [4]. However, IAP coverage is incomplete even in the best of settings. Furthermore, the burden of invasive GBS disease may be high in resource poor countries such as Africa (estimated incidence of 1.12/1000 live births) where IAP implementation is challenging [5]. Concerns have arisen as to the possible negative impact of large-scale prevention, as IAP may promote the emergence of antibiotic resistance, and early exposure to antibiotics can disrupt the development of the intestinal microbiome, with consequences in adulthood [6]. Finally, IAP has no impact on LOD, stillbirths and prematurity due to GBS, as well as a limited impact on disease in pregnant women [7]. Further strategies are urgently required to decrease GBS-associated morbidity and mortality.

There are ten known GBS serotypes (Ia, Ib and II-IX), but serotype Ia, III and V are more commonly responsible of invasive GBS disease in infants under 90 days of life. Multivalent vaccines administered to pregnant women to protect their infants against GBS disease could overcome many of the outstanding issues related to IAP and could be an effective strategy for resource-poor countries. Indeed, compared to WHO European region, in WHO African region mortality rates are 7 times higher (7/1000 vs 51/1000 LBs) [8].

Therefore, the prevention of neonatal infections through maternal immunoprophylaxis is a topic that has recently aroused wide attention. The purpose of this strategy is to induce maternal protective immunity resulting in a specific transplacental IgG passage. Indeed, recent data have shown that vaccinating pregnant women does not increase adverse events or fetal risks [9]. WHO data from developing countries show a 92% decline (from the 1980s to 2000) in neonatal tetanus case fatalities following maternal vaccination with tetanus toxoid [10].

It is estimated that to detect a 75% reduction in EOD and LOD in countries with a disease incidence of more than 1/1000 births it would be necessary to enroll about 60,000 pregnant women to study the effectiveness of the vaccine, assuming that this protects from 90% of circulating serotypes [11]. Therefore, in order to facilitate the licensure of a vaccine, the study of protective serocorrelates, followed by a demonstration of a post-license efficacy, aroused interest. Although previous studies have shown an association between serotype-specific maternal IgG titers and reduction of neonatal disease risk, no study has been able to establish with certainty a protective antibody threshold value, due to different assays for determining antibody titers or inability to compare and pool the results of different studies [12]. Vaccines have been tested against serotype-specific capsular polysaccharide and against surface proteins that are expressed in different serotypes and could then protect against specific serotypes [7, 12].

PREPARE is a non-profit, multi-center, interventional and experimental study. It aims to develop a maternal vaccine platform in Uganda, determine pregnancy outcomes and to define the extent of GBS infections in children and mothers in a sub-Saharan context. It also aims to estimate the protective serocorrelates against the main GBS serotypes that cause diseases in Europe and Africa and to conduct two trials on candidate GBS vaccines. The PREPARE project involves 7 countries across the world (Malawi, Uganda, South Africa, the United Kingdom, the Netherlands, Italy and France) and aims to develop a serum biobank, in order to define serocorrelates of protection against GBS, by using standardized antibody assays and a bacterial strains bank to study the characteristics of neonatal and maternal strains.

The overarching objectives will be achieved through 6 work packages (WPs), each with specific aims (Table 1). Italy (that belongs to WP3) is represented by a network made up of 41 centers across the country (Table 2), coordinated by the Azienda Ospedaliero-Universitaria Policlinico (Modena). The Italian network will collect at least 50 neonatal GBS invasive cases (defined as an infant with isolation of GBS from blood culture or from culture of cerebro-spinal fluid) within 2 years. Strains will be sent to the national referring center (Istituto Superiore di Sanità) for GBS typing.

**Table 1:** Work-Packages (WP) of the PREPARE Study: role, goals and participating countries

Work packages	Role in the project	Goals	Participating country
**WP1**	Project Management, Scientific Coordination and Oversight of Capacity Building**.**	To ensure conduct of all research activities to the highest standards, including oversight and coordination of the other WPs to ensure deliverables and milestones are met.	United Kingdom South Africa
**WP2**	Clinical trial site development and GBS disease surveillance.	To establish the GBS disease incidence in an urban Ugandan cohort followed to 3 months of age and establish the baseline rates of common obstetric and neonatal outcomes in preparation for WP4 and WP5.	Uganda South Africa United Kingdom
**WP3**	Developing serocorrelates of protection against GBS	To develop a biobank of prospectively collected sera from cases of GBS disease and associated GBS disease isolates from a network of African and European sites in order to define serocorrelates of protection against GBS, using standardized antibody assays.	Uganda, Malawi, South Africa, United Kingdom, **Italy**, France, Netherlands
**WP4**	Multivalent CPS-conjugate vaccine trial.	To conduct a phase II study of a multivalent vaccine against the GBS CPS in pregnant HIV-infected and uninfected women and to establish a platform for future trials with new GBS vaccines.	United Kingdom Uganda
**WP5**	Minervax Alp-NN GBS vaccine trial.	To conduct a phase II study of a multivalent vaccine against the Alp and Rib proteins on the surface of GBS in pregnant HIV-infected and uninfected women and to establish a platform for future trials with new GBS vaccines.	Denmark United Kingdom South Africa
**WP6**	Communications, networking and dissemination.	To develop a strategy for patient and public involvement, communications, capacity strengthening and stakeholder engagement.	United Kingdom

*CPS* Capsular polysaccharide, *GBS* group B streptococcus

**Table 2:** Partner of the PREPARE Consortium

Organisation	Country	Role	H2020 type of organisation
St George’s Hospital Medical School (SGUL)	United Kingdom	Coordinator	Secondary or higher education establishment
Makerere University - Johns Hopkins University Care Ltd	Uganda	Participant	Research organisation
University of Liverpool	United Kingdom	Participant	Secondary or higher education establishment
Wits Health Consortium (PTY) LTD	South Africa	Participant	Secondary or higher education establishment
Assistance Publique Hopitaux de Paris (AP- HP)	France	Participant	Secondary or higher education establishment
Academisch Medisch Centrum bij de Universiteit van Amsterdam	The Netherlands	Participant	Secondary or higher education establishment
Azienda Ospedaliero- Universitaria di Modena	Italy	Participant	Secondary or higher education establishment
University College London (UCL)	United Kingdom	Participant	Secondary or higher education establishment
London School of Hygiene and Tropical Medicine (LSHTM)	United Kingdom	Participant	Secondary or higher education establishment
Minervax ApS	Denmark	Participant	Small or medium sized entreprise
Pfizer Inc.	United Kingdom	Participant	International Organisation

The PREPARE Italian network will collect: i) isolates from infants with invasive disease (cases), together with maternal and neonatal sera collected at the time of diagnosis of infant disease; ii) cord sera and GBS strains (of the same serotype as cases) from colonized mothers whose infants do not develop GBS infection (controls). Biological materials will be used for i) determining the concentration of specific IgG anti-GBS (serotype III the most frequent cause of neonatal disease) in the cord serum of healthy controls and in the serum of infants (aged 0 to 90 days of life) with GBS infection ii) assessing a correlation between antibody concentration and GBS disease risk and iii) validating estimates of protective serocorrelates.

## Conclusions

Despite the progress made in high-income countries in the prevention of EOD, GBS remains an important cause of morbidity and mortality in the first months of life worldwide.

A maternal GBS vaccine could reduce the burden of both EOD and LOD, maternal puerperal sepsis, stillbirth and preterm delivery. A vaccine could help to overcome the inherent limitations of IAP, and could reduce unnecessary antibiotics, as well as costs and long-term disabilities consequent to GBS infection. Finally, a vaccine could be an effective strategy for resource-poor countries, where the antenatal screening and large-scale IAP might be unfeasible. PREPARE aims to undertake clinical trials of a maternal GBS vaccine, to determine pregnancy outcomes, and to estimate the protection serocorrelates against the main GBS serotypes that cause diseases in Europe and Africa.

## Data Availability

Not applicable.
